# Hydrophobins: multifunctional biosurfactants for interface engineering

**DOI:** 10.1186/s13036-018-0136-1

**Published:** 2019-01-23

**Authors:** Bryan W. Berger, Nathanael D. Sallada

**Affiliations:** 1 Department of Biomedical Engineering, University of Virginia, Thornton Hall, P.O. Box 400259, Charlottesville, VA 22904 USA; 2 Department of Chemical Engineering, University of Virginia, 214 Chem. Eng., 102 Engineers’ Way, Charlottesville, VA 22904 USA

## Abstract

Hydrophobins are highly surface-active proteins that have versatile potential as agents for interface engineering. Due to the large and growing number of unique hydrophobin sequences identified, there is growing potential to engineer variants for particular applications using protein engineering and other approaches. Recent applications and advancements in hydrophobin technologies and production strategies are reviewed. The application space of hydrophobins is large and growing, including hydrophobic drug solubilization and delivery, protein purification tags, tools for protein and cell immobilization, antimicrobial coatings, biosensors, biomineralization templates and emulsifying agents. While there is significant promise for their use in a wide range of applications, developing new production strategies is a key need to improve on low recombinant yields to enable their use in broader applications; further optimization of expression systems and yields remains a challenge in order to use designed hydrophobin in commercial applications.

## Introduction

**Fig. 1:**
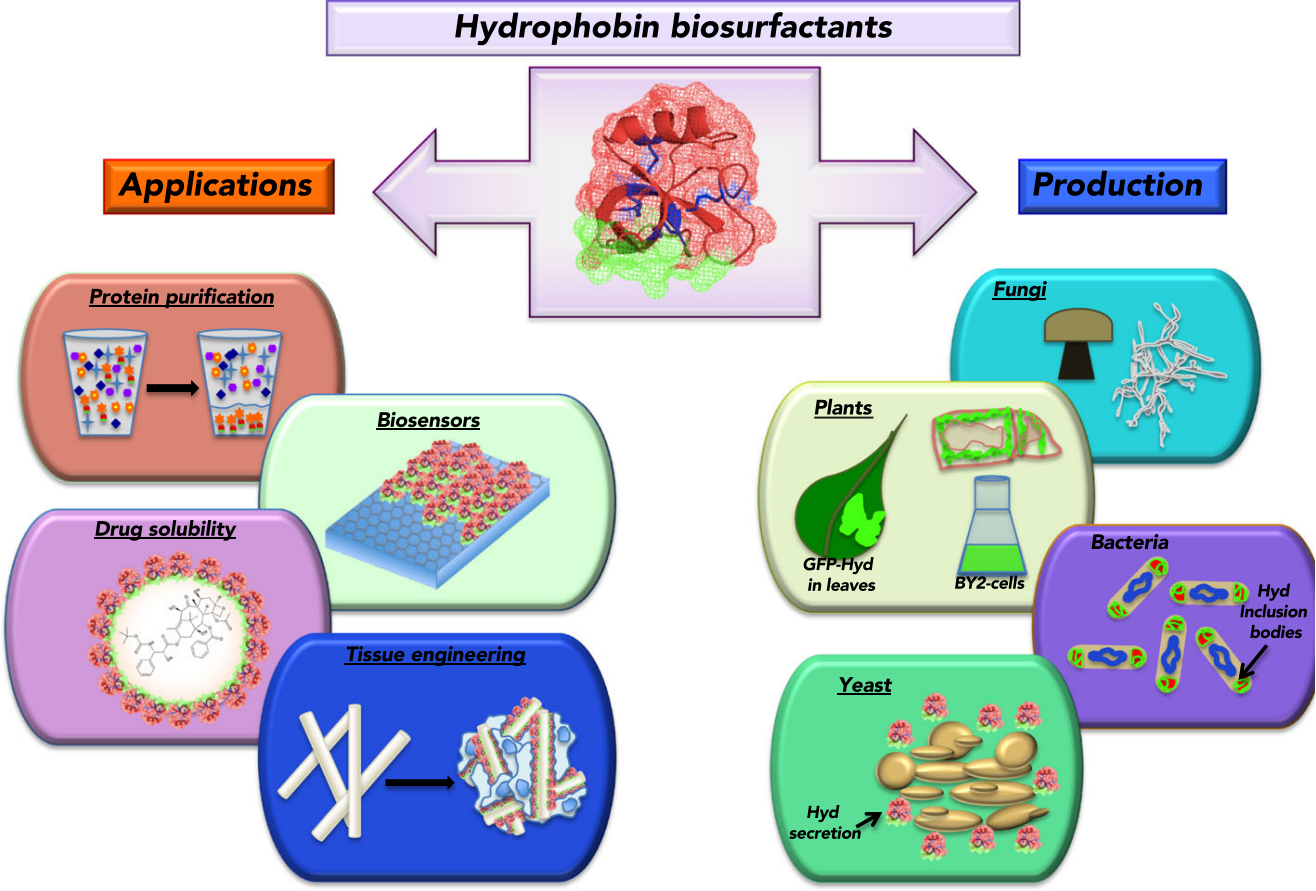
Visual summary of current hydrophobin applications and production systems

Hydrophobins are a family of small (< 20 kDa), highly surface-active globular proteins that play diverse roles in filamentous fungi growth and development [1–3]; they have been cited as the most surface-active proteins known [3]. Structurally, hydrophobins are characterized by the presence of 8 highly conserved cysteine residues in a specific primary sequence pattern, forming 4 disulfide bonds [4–9]. These 4 disulfide bonds stabilize an amphipathic tertiary structure which imparts surfactant-like activity [5, 6, 10, 11], driving hydrophobin self-assembly into amphipathic layers at hydrophobic-hydrophilic interfaces. Hydrophobins have historically been separated into two groups, class I and class II, based on their hydropathy plots, solubility characteristics, and structures formed during self-assembly [12, 13]. Specifically, class I hydrophobins, like SC3 from *Schizophyllum commune*, form highly insoluble amyloid-like rodlets at interfaces [2, 8, 14, 15], often proceeding through a conformational change [14–16], that can only be dissolved using strong acids [17, 18]. In contrast, class II hydrophobins, like HFBI or HFBII from *Trichoderma reesei*, form a highly ordered 2D crystalline monolayer at interfaces [19–21] that can easily be dissolved with detergents, organic solvent solutions, or high pressure [3]. Interestingly, the structural and functional roles of the conserved disulfide bonds differ between the two classes, with disulfides of class I hydrophobin SC3 being necessary to keep the protein soluble and structurally stable, but not affecting the self-assembling ability [10], while class II hydrophobin HFBI disulfides are critical to both protein structure and stability as well as function at interfaces [11]. Recently, intermediate hydrophobin types have been discovered that are between class I and class II either structurally [22, 23] or functionally [24, 25].To this end, *Lo* et al showed that by producing genetic chimeras of class I hydrophobins EAS and DewA with class II hydrophobin NC2, properties of both classes of hydrophobins could be obtained [21]. The high sequence diversity within the hydrophobin family suggests multiple biological roles of these biosurfactants [18, 26, 27], with complementation studies suggesting, at least for class I hydrophobins, that each was evolved to function at a specific interface [3], which is also demonstrated by the differential expression and localization of different hydrophobins during *S. commune* development [28]. Recently, Pham et al determined that hydrophobins have a significant level of conformational plasticity, with the nature of the interfacial assemblies being highly dependent on the specific interface the proteins are interacting with [29]. Lienemann et al found that by engineering native surface charges on hydrophobin HFBI, viscoelastic properties of the assembled film at the air-water interface and ability to absorb secondary protein layers were affected [30]. Additionally, Meister et al showed that mutating the surface charges of HFBI does not affect overall protein folding state, but specific charge mutations could be linked to inter-protein interactions at the assembled film, while other mutations were linked to protein orientation at the interface [31]. Meister et al also reported that HFBI adsorbed to the air-water interface reoriented in a pH responsive way due to changes in inter-protein interactions caused by side-chain charge states [32]. Overall, these findings indicate a significant potential to use hydrophobins both directly and in modified forms for many interface-engineering applications, which will be the primary focus of this review. For other areas of active hydrophobin research such as foam stabilization and gushing, the reader is referred to the following reviews [33, 34]. Additionally, the current hydrophobin production modalities and pitfalls will be discussed (Fig. 1).

## Applications

The vast diversity among known hydrophobins, the specificity of particular hydrophobins in their roles in fungal development, and their unique structures and surface activity implicate hydrophobins as advantageous agents in many applications where interfaces need to be altered, bridged, or stabilized.

In biomedical applications, hydrophobins have been particularly useful for hydrophobic drug formulation and delivery. While hydrophobic drugs are often formulated using surfactants such as Tween 80 or Chremophore EL to improve their solubility in an aqueous environment, these surfactants are not innocuous, and have been shown to be immunogenic in immunocompromised patients, such as cancer patients [35]. Aimanianda et al showed that hydrophobins forming the hydrophobic rodlet layer of airborne fungal spores are responsible for the immunological silencing that occurs when a host breathes the spores [36], which suggests that hydrophobins have the opposite effect of industrial surfactants on the immune system, and may act as an immune-suppressive barrier in drug formulations.

Given their high surface activity, hydrophobin-based drug stabilization has been an area of active research [37–42]. Valo et al demonstrated the preparation of class II hydrophobin-coated drug nanoparticles below 200 nm that were stable for at least 5 h in suspension, and for longer times after freeze-drying [37]. They also utilized a hydrophobin fused to green fluorescent protein (GFP) to demonstrate that the particles were indeed decorated with the proteins, and suggested that hydrophobin fusions could be used to further modify the particle surfaces [37]. Hydrophobin HFBI produced as a genetic fusion to cellulose binding domains allowed a cellulose-based nanofibrillar matrix stabilization of hydrophobin stabilized drug particles of around 100 nm, capable of over 10 months storage and enhanced drug dissolution rates [38]. Sarparanta et al. showed that functionalizing thermally hydrocarbonized porous silicon nanoparticles with hydrophobin HFBII altered the biodistribution compared to unfunctionalized particles, as well as altered the protein adsorption profile to the particle surface [39]. Fang et al utilized a commercially available surfactant blend containing class I hydrophobin, H star protein B [43], to solubilize the chemotherapy drug docetaxel [41]. They showed that the formulation was biocompatible and exhibited a high drug loading, high nanoparticle yield, small particles of narrow distribution, and delayed drug release in rats [41]. Moreover, the effective stabilization of model drug oil-in-water emulsions using low concentrations of HFBII with nanofibrillar cellulose suggests an additional advantage of formulation with hydrophobins since less material is needed compared to traditional pharmaceutical surfactant-based emulsion stabilizers [42]. When the class I hydrophobin SC3 was used to solubilize the hydrophobic drugs cyclosporine A and nifedipine, the oral bioavailability was increased by 2- and 6-fold, respectively [44]. Hydrophobins have also been explored, with positive results, as a topical drug formulation agent for nail permeation [45, 46]. Thus, several drug-formulations and administration modalities implicate hydrophobins as effective adjuvants for improved hydrophobic drug solubility, stability, and bioavailability. Furthermore, by using a protein-based biosurfactant capable of manipulation at the genetic level, hydrophobin fusion proteins have also been employed for specific drug targeting. Recently, Reuter et al demonstrated that coating porous silicon nanoproteins with a fusion of *T. reesei* class II hydrophobins to human transferrin protein resulted in their uptake in cancer cells [47]. Also, the stabilizing effect of the highly conserved disulfide bonds in class II hydrophobins has been exploited as a drug-release mechanism [48]. Maiolo et al used the class II hydrophobin HFBII to organize and stabilize supraparticles of dodecanethiol-protected gold nanoparticles that could be loaded with hydrophobic drug and remain stable in the blood until taken up by tissues, where cytoplasmic glutathione would reduce the disulfides allowing the supraparticles to release the drug load directly in the cytoplasm [48]. This resulted in a two orders of magnitude enhancement of the anticancer drug therapeutic efficiency [48]. Overall, these studies show the feasibility of hydrophobin-based drug formulation and point to a need to continue to understand hydrophobin structure and function as a means to engineer novel hydrophobins for biocompatible coatings that improve both drug bioavailability and targeting.

The self-assembly characteristics of hydrophobins renders them conducive to biosensor applications as well. Corvis et al used class I hydrophobin coating from *S. commune* to render glassy carbon electrodes catalytic by immobilizing redox enzymes to the hydrophobin layer [49]. Also, Zhao et al utilized class II hydrophobin HFBI as an enzyme immobilization matrix on platinum electrodes to create a selective and efficient glucose biosensor [50]. Later, HFBI was used to alter the surface wettability of a gold surface and immobilize the enzyme choline oxidase [51]. They found that the HFBI layer could withstand pH values from 1 to 13, and was able to behave as an amperometric choline biosensor, further suggesting the potential of hydrophobins in electrochemical biosensing applications [51]. After 7 weeks of storage, the sensor retained > 70% of its initial activity, suggesting the stability of the protein film [51]. More recently, Spadavecchia et al reported that by using gold nanoparticles complexed with a class I hydrophobin Vmh2, which has a natural propensity to bind carbohydrates, a glucose biosensor could be generated using a one-pot synthesis approach [52]. This introduces the idea of using specific hydrophobins with unique and intrinsic biological properties on an application-specific basis. Similarly, a class I hydrophobin-based biosensor for small peptides, specifically yeast pheromones, was reported that enabled an extremely low limit of detection by using combinations of alpha-factor labeled and unlabeled hydrophobins [53]. Recombinant class I EAS hydrophobin was expressed in *E. coli* with and without the yeast alpha factor, and used to wet a hydrophobic polystyrene surface [53]. The researchers found these biosensors were robust against changes in the sample composition, and due to the high stability of the hydrophobin monolayer, as it was able to withstand hot 2% sodium dodecyl sulfate (SDS) extraction from the polystyrene surface, they could be reused several times without loss of sensitivity [53]. Soikkeli et al designed class II hydrophobin HFBI fusion proteins fused to Protein A or a small peptide Z_E_ produced either in plant or fungal systems to create graphene biosensors that are label free and have femptomolar sensitivities with approximately 1 s readout [54]. The biosensors could be prepared in one-step due to the self-assembling nature of the hydrophobin domain in the fusion proteins, and demonstrated that the receptor modules could be removed and replaced with a different receptor module in situ [54]. Further, they showed that the monolayers survive drying, indicating a reasonable shelf life, and showed that both large and small analytes (immunoglobin and charged peptide) are compatible with this system [54]. In yet another interesting sensor-related application, genetically modified hydrophobin HFBI with an N-terminal cysteine residue were used to selectively nanopattern gold-nanoparticles onto a hydrophobic surface in a pH controlled manner [55]. This allowed production of nanoscale components with a functional electronic interface [55]. The hydrophobin HFBI was also used in a method to exfoliate and functionalize graphene sheets [56].

The surface activity and self-assembly of hydrophobins suggest a broad and growing potential application space. Some additional applications include hydrophobins used as protein purification tags [57–60], protein and cell immobilization [61–65], antimicrobial coatings [66], and biomineralization [67, 68]. Linder et al first demonstrated that class II hydrophobins from *T. reesei* could be efficiently separated in aqueous two phase systems (ATPS) using nonionic surfactants from crude fungal culture supernatants, and then efficiently back extracted using isobutanol with a partition coefficient over 2500 for HFBI [57]. Joensuu and colleagues later utilized this separation technology to purify Green Fluorescent Protein (GFP)-HFBI fusion expressed in *Nicotiana benthamiana* leaves, and reported enhanced accumulation of GFP in the leaves due to formation of novel protein bodies, as well as a 91% selective recovery of the GFP-HFBI fusion at concentrations of 10 mg ml^− 1^ after ATPS separation [58]. Reuter et al explored other class II hydrophobin fusion partners to GFP in the same system and found that efficiency of separation was highly hydrophobin dependent, with only two of the eight new hydrophobins efficiently concentrating GFP to the surfactant phase from plant extracts [59], which suggests specific molecular determinants of separation efficiency. Hydrophobin ATPS separation has also been used to indirectly capture proteins with affinity for the hydrophobin fusion partner. Recently, an HFBI fusion to Protein A, an antibody binding protein, was produced recombinantly in both *N. benthamiana* leaves and tobacco BY-2 suspension cells, then utilized in a nonionic surfactant ATPS to efficiently bind and purify antibodies in solution [60].

Hydrophobins have also been successfully applied to reversing the wettability of poly(dimethylsiloxane) (PDMS), a material commonly used in microfluidic devices. Wang et al showed that PDMS could effectively be turned from hydrophobic to hydrophilic using a hydrophobin surface layer, which then rendered the surface bioactive so that antigen molecules could be patterned onto the surface layer [61]. Washing the PDMS with water did not remove the stability deposited hydrophobin films from the surface [61]. Hou and colleagues explored the class I hydrophobin HGFI on PDMS wettability, and found that it had higher stability in this application than class II hydrophobin HFBI, able to withstand washes with hot 2% SDS [62]. Furthermore, the versatility of hydrophobin HFBI as a protein-immobilization layer on both hydrophobic and hydrophilic substrates was demonstrated by Qin et al, whereby adsorbed HFBI layers on both PDMS (hydrophobic) and mica (hydrophilic) could immobilize chicken IgG for biosensing applications [63]. They noted that the modified water contact angle due to hydrophobin deposition did not change when the surfaces were stored for several days in air or water, indicating the hydrophobins were stable in this configuration [63]. Similarly, Zhang et al used hydrophobin HFBI to improve hydrophilicity and design bioactive surfaces of electrospun PCL grafts used in tissue engineering [64]. Anti-CD31 antibody could then be immobilized to the PCL surface through the HFBI layer, which promoted the attachment and retention of endothelial cells to the graft [64]. Enhanced cellularization and vascularization of PCL scaffolds was similarly accomplished using a vascular endothelial growth factor fused to class I hydrophobin HGFI produced in the yeast *Pichia pastoris* [69]. Additionally, Boeuf et al exploited a recombinant class I hydrophobin DewA fused to an integrin binding Arginine-Glycine-Aspartic acid motif (RGD) or laminin domain to enhance adhesion of mesenchymal stem cells, osteoblasts, fibroblasts, and chondrocytes to orthopaedic implant surfaces without affecting the propensity of the bacteria *Staphylococcus aureus* to adhere [65]. To generate explicitly antibacterial surface coatings, class IIa bacteriocin pediocin PA-1, an antibacterial peptide, was expressed in *Saccharomyces cerevisiae* fused to the class I hydrophobin HGFI and used to functionalize and greatly improve the bacterial resistance of electrospun PCL grafts [66].

In biomineralization applications, Heinonen et al engineered hydrophobin HFBII modified with a ceramophilic protein sequence to mineralize calcium carbonate [67]. The microparticles produced were uniform and exhibited amphiphilic properties that were demonstrated by preparing pickering emulsions [67]. Melcher et al used a modified class I DewA hydrophobin fusion in a biomimetic approach to enhance hydroxyapatite binding and calcium phosphate nucleation for reconstruction of eroded teeth [68].

In an additional application, Taniguchi et al have used commercially available class I hydrophobin H*protein B [43] in a ligand encapsulation process to phase transfer quantum dots from solvent to aqueous phases. They demonstrated that encapsulating quantum dots allowed for efficient phase transfer while maintaining a significant portion of emission characteristics, and allowing for additional conjugation for biological imaging applications [70]. HFBI fused to an RGD motif was recently employed as a solubilizing agent for a hydrophobic boron-dipyrromethene (BODIPY) dye, with the RGD motif on the encapsulated dye causing effective labeling of tumors in nude mice [71].

## Production of hydrophobins

While hydrophobin research has ballooned over the last two decades and application space is growing in terms of impact and diversity, the commercial viability of hydrophobins has been hampered by generally low yields.

Askolin et al were able to overproduce the class II hydrophobin HFBI by homologous expression in *T. reesei* using a clone with 3 copies of the HFBI gene to a production level of 600 mg L^-1^ [72]. However, most of the hydrophobin (80%) was bound to the mycelium and required further extraction steps to obtain pure protein [72]. In trying to overproduce the class I hydrophobin SC3 via homologous expression in *S. commune* using multiple gene copies, Schuurs et al observed gene silencing of the endogenous and introduced SC3 genes at the transcriptional level due to gene methylation [73]. Turning to heterologous production of SC3 in *T. reesei* yielded approximately the same level of SC3 as the native *S. commune* [74]. Thus, recombinant production, using either prokaryotic or eukaryotic organisms, has been an attractive choice to try to overproduce both native type or engineered hydrophobins as a means to enhance scalability and avoid pitfalls of using the homologous host. In bacteria, however, hydrophobin production, especially for class I hydrophobin, has typically been on the order of 10 to 100 mg L^− 1^, but often less [75–78]. In many cases, recombinant hydrophobin production in bacteria proceeds through purification from inclusion bodies, requiring a denaturation/renaturation step to achieve the final product [8, 53, 76, 77, 79]. These denaturation and refolding steps represent added expense for large-scale hydrophobin production using these heterologous systems. On rare occasions for specific hydrophobins these pitfalls were overcome, as for the case of H star A and B proteins where advantageous fusions and expression conditions have yielded industrially feasible amounts of soluble class I hydrophobin from bacteria [43]. More recently, however, eukaryotic heterologous expressions systems have been employed to greatly increase yields of both class I and class II hydrophobins recombinantly with generally better yields than bacteria.

The methylotropic yeast *Pichia pastoris* has become a popular heterologous host for hydrophobin expression [11, 69, 80–86]. *P. pastoris* has several advantages for heterologous hydrophobin production. As a fungal host, *P. pastoris* is expected to share similar chaperone proteins and folding strategies as filamentous fungi. These include glycosylation and proper disulfide bond formation [87, 88], which has been shown to be critical in class II hydrophobin structure and function [11] as well as to class I hydrophobin stability [10]. In addition, recombinant proteins can be secreted into the culture medium of *P. pastoris* [89], which secretes very low levels of endogenous proteins, under the control of a highly inducible promoter such as the methanol induced AOXI promoter [89, 90]. This means the recombinant proteins are effectively pre-purified by being secreted [89]. In particular, Niu et al have been able to express class II hydrophobin to levels of 120 mg L^− 1^ in *P. pastoris* [81], while class I hydrophobins RodA and RodB as well as HGFI were produced to levels of between 200 and 300 mg L^− 1^ [83, 85]. This represents a substantial improvement to previously reported yields but could be further improved with additional optimization. Of interest is the reported increase in HGFI production in *P. pastoris* from shake flasks yielding 86 mg L^− 1^ [82] to fed-batch fermentation yielding 300 mg L^− 1^ [85]. By optimizing the process parameters using controlled feed rate in the fed-batch fermentation, the recombinant class I hydrophobin HGFI yield was increased over 3-fold between these studies. Of note is the possibility for hydrophobin producing *P. pastoris* strain optimization at the molecular level [87] to further increase yields at an intrinsic level in conjunction with extrinsically optimized growth conditions, such as growth media pH and composition, temperature, and feed rate. Molecular optimizations might be to increase strain copy number of the target gene, which has been shown to often correlate to higher product expression in *P. pastoris* [91].

The other emerging heterologous hosts in hydrophobin production are plant based. Transient or stable expression of GFP-HFBI fusion has been carried out in *Nicotiana benthamiana* leaves [58, 59, 92] and tobacco BY-2 suspension cells [93, 94] to produce high yields of hydrophobins and hydrophobin fusions. In particular, Joensuu et al reported a GFP-HFBI production level of 3.7 mg g^− 1^ fresh leaf weight [58], comprising approximately 51% of the total soluble protein. Häkkinen et al recently reported the yield of a high-expressing BY-2 clone as 1.1 g L^− 1^ of GFP-HFBI in suspension, and also reported successful cryopreservation of the cultures, enabling industrial application of this hydrophobin fusion production technology [94]. The interesting development of hydrophobin-induced protein bodies in the leaf cells were credited with the increased accumulation of the recombinant proteins, keeping them in a protected state from proteases in the cytosol [58, 60, 92].

## Conclusions and future perspectives

The remarkable surface-activity of hydrophobins has made them attractive candidates in a wide variety of interface-engineering applications to date. While some very specific hydrophobins can be made at industrially feasible levels, there remains an unmet need to produce high levels of both native and engineered forms of hydrophobins before hydrophobin-based technologies can fully realize their commercial potential. Furthermore, a deeper understanding of hydrophobin structure-function relationships would inform novel hydrophobin design for specific applications, which would have tremendous implications in many important fields such as pharmaceuticals, electronics, microfluidics, and food products. To date, biochemical studies have related stability to disulfide bonds [10, 11] and film viscoelastic properties, inter-protein interaction, and pH responsive orientation to surface charge [30–32]. However uncovering other key structure-function relationships in hydrophobins could lead to design from first principles, whereby application specific characteristics could be programmed into the hydrophobin at a genetic level to enable outcomes such as increased binding, enhanced solubilization, switchable surface activity, or specific nanopatterning, although the potential is limitless. To get at this goal, further research into hydrophobin sequence, folding, and the related function needs to be undertaken in order to build a foundation for design.

## Abbreviations

ATP: Aqueous two phase systems
GFP: Green fluorescent protein
PDMS: Poly(dimethylsiloxane)
